# Genetic Variations of Cytokines and Cytokine Receptors in Psoriasis Patients from China

**DOI:** 10.1155/2014/870597

**Published:** 2014-05-25

**Authors:** Xiao-Lan Li, Chun-Feng Wu, Gui-Sheng Wu

**Affiliations:** ^1^Department of Dermatology and Rheumatology, Affiliated Yan'an Hospital of Kunming Medical College, Kunming, Yunnan 650051, China; ^2^State Key Laboratory of Phytochemistry and Plant Resources in West China, Kunming Institute of Botany, The Chinese Academy of Sciences, Kunming, Yunnan 650201, China

## Abstract

Psoriasis is a chronic inflammatory and hyperproliferative skin disease affected by both genetic and environmental factors. The aim of the present study was to investigate polymorphisms in a candidate gene family of interleukin (IL) in unrelated Chinese patients with psoriasis and control subjects without psoriasis. In this case-control study, 200 unrelated Chinese psoriasis patients and 298 age- and sex-matched control subjects were enrolled. Genomic DNA was prepared from peripheral blood obtained from all psoriasis patients and control subjects. We genotyped seven single-nucleotide polymorphisms (SNPs) in candidate genes of six ILs: IL4, IL10, IL12B, IL13, IL15, and IL23R, which have been shown in the literature to be associated with psoriasis in other ethnic groups. Among the seven SNPs in the six IL genes studied, only the rs3212227 in the IL12B gene was found to be associated with psoriasis at genotypic level in the studied population. The C/C genotype in the IL12B gene is a protective factor of psoriasis (*P* = 0.0218; OR = 0.51; 95% CI: 0.27–0.96) in Chinese. Furthermore, the studied Chinese population has extremely low minor allele frequency for IL23R. Together, the data reveal unique genetic patterns in Chinese that may be in part responsible for the lower risk for psoriasis in this population.

## 1. Introduction

Psoriasis is an immunologically mediated chronic inflammatory and hyperproliferative skin disease affected by both genetic and environmental factors. The prevalence of psoriasis varied among populations with different genetic backgrounds and habitats, from 3% in Northern Europe and 2% in North America and the UK to 0.1–0.3% in American Indians and East Asia [1, 2]. Psoriasis is proposed to be associated with other immune diseases, such as arthritis and Crohn's disease [3]. Initial causative research has identified strong association between psoriasis and the interleukin genes (IL4, IL10, IL12B, IL13, and IL23R) in the northern European from US and UK [4–7]. Furthermore, the SNP rs56245420 in the IL15 gene has been found to be associated with psoriasis in the Chinese Han population but not in any of the UK, German, or US Caucasian populations investigated [8–10], since the minor allele frequency for this SNP and others across IL15 differs quite strikingly between the populations, suggesting heterogeneity in the genetic susceptibility to psoriasis.

In this study, we aimed to determine if psoriasis is associated with six IL genes that have been strongly associated with psoriasis in Europeans but not well studied in Chinese. The seven included SNPs are rs2243250 in the IL4 gene, rs1800872 in the IL10 gene, rs3212227 in the IL12B gene, rs1800925 and rs20541 in the IL13 gene, rs56245420 in the IL15 gene, and rs11209026 in the IL23R gene.

## 2. Materials and Methods

### 2.1. Study Population

A total of 200 psoriasis patients and 298 healthy controls were recruited in this study (see Table 2). All participants did not suffer from any other diseases and belonged to Han nationality in Yunnan Province, China. The study was performed according to the Helsinki Declaration with approval of the institutional review boards of the Affiliated Yan'an Hospital of Kunming Medical College and the Kunming Institute of Botany. Informed consent was obtained from each participant before inclusion in this study.

### 2.2. Determination of Genotype

Genomic DNAs were isolated from whole blood using regular phenol/chloroform method. The SNP rs11209026 in the IL23R gene was genotyped by the TaqMan allelic discrimination method (Applied Biosystems). New PCR-RFLP methods were generated to genotype the SNP rs56245420 in the IL15 gene. Primers 5′-TTT CTG TTA TTA ACA AAC ATC ACT CTG-3′ and 5′-CAA CAC TTG TAC ATA TTT TTA TTC** AAt AT**-3′ (mismatch is shown in bold lower case) were used for rs56245420. Other five SNPs were genotyped by PCR-RFLP methods described previously with slight modification [11–15]. PCR reaction was carried out in a total volume of 20 *μ*L containing 20 ng of genomic DNA, 1 × PCR buffer, 1.5 mM MgCl_2_, 200 *μ*M of each dNTP, 30 ng of each primer, and 1 unit of Taq DNA polymerase (TakaRa). Samples were denatured at 95°C for 2 min followed by 30 cycles of 94°C for 45 sec, 61°C (rs2395029) or 54°C (rs56245420) for 45 sec, and 72°C for 45 sec and ended with a final extension for 7 min at 72°C. PCR products were digested with 4 U of appropriate restriction endonuclease and electrophoresed on 3% agarose gels and stained with ethidium bromide. The restriction endonucleases, PCR product lengths, and restriction patterns are shown in Table 1.

### 2.3. Data Analysis

Statistics analysis was performed by SPSS software for windows (SPSS Inc.). The frequencies of genotypes and alleles for all the six studied loci were determined assuming codominant inheritance. The Hardy-Weinberg equilibrium (HWE) for six loci in psoriasis patients and controls was tested by means of chi-square tests. The statistical significance of the genotype and allele frequency variables between the psoriasis patients and control group was evaluated by chi-square test with Yates correction for small numbers. Relative risk associated with the significant genotype was estimated by the odds ratio (OR). OR with 95% confidence intervals (95% CI) was tested using a chi-square distribution and the null hypothesis being tested is OR = 1. *P* values <0.05 were considered as statistically significant.

## 3. Results

Only one of the fourteen Hardy-Weinberg tests (seven polymorphic loci each in the psoriasis patient and control groups) had *P* values smaller than 0.05 (*P* = 0.01 at rs3212227 in controls). All nine remaining genotype frequencies fit Hardy-Weinberg expectations according to chi-square tests in psoriasis patients and controls (*P* > 0.05). Therefore, there is no meaningful deviation from WHE, and our population is derived from random mating.

Polymorphism (minor allele frequency > 1%) has been found for all studied SNPs except for rs11209026 in the IL23R gene (Table 2). Table 2 shows that rs3212227 in the IL12B gene (*P* = 0.0218) was associated with psoriasis at genotypic level in the studied population. Other SNPs examined were not associated with psoriasis considered from single locus. As shown in Table 3, while the A/C genotype (OR = 1.48; 95% CI: 0.95–2.30) and the alleles (OR = 0.84; 95% CI: 0.63–1.13) at rs3212227 in the IL12B were not a risk factor of psoriasis, the C/C genotype was a protective factor of psoriasis (OR = 0.51; 95% CI: 0.27–0.96).

## 4. Discussion

The etiology of psoriasis is a complex interaction of environmental and biological factors. Genetic factors may play a significant role in the risk of psoriasis in Chinese [10, 16]. Recent genetic studies indicate that the location of these genes varies considerably among populations and families. We are interested to know if psoriasis is associated with the genes that have been strongly associated with psoriasis in Europeans but not well studied in Chinese.

Our results showed that the IL12B gene was associated with psoriasis in Chinese at genotypic level (*P* < 0.05), which is in line with the findings from European studies [17]. The C/C genotype for rs3212227 in IL12B is a protective factor (OR = 0.51) from psoriasis. Similar result from studying SNP rs6887695 in IL12B showed that the minor allele C was a protective factor from psoriasis [5].

The nonsynonymous SNP in IL23R, rs11209026, widely thought to be the primary psoriasis-associated SNP in IL23R in Europeans, was found not to be polymorphic in Chinese, which is in agreement with the findings of others [18]. The low frequencies of variant in IL23R are accordingly of low risk for psoriasis in Chinese. With single SNP analysis, no association is found between the psoriasis and the IL4, IL10, IL13, and IL15 genes.

## Figures and Tables

**Table 1:** Primer sequences, PCR product lengths, restriction endonucleases, and restriction patterns for the PCR-RFLP analyzed SNPs.

SNP	Gene	Position of SNP in genomic sequence	Forward primer^a^	Reverse primer	Anneal temperature (°C)	Product length (bp)	Restriction endonucleases	Fragments of frequent allele genotype (bp)	Fragments of heterozygous genotype (bp)	Fragments of rare allele genotype (bp)	Reference
rs2243250	IL4	132009154:C/T	TAAACTTGGGAGAACAT**G**GT	TGGGGAAAGATAGAGTAATA	49	195	AvaII	195	22 + 173 + 195	22 + 173	[1]
rs1800872	IL10	206946407:A/C	AGGTGATGTAATATCTCTGT	TAAATATCCTCAAAGTTCC	57	303	RsaI	65 + 238	65 + 238 + 303	303	[2]
rs3212227	IL12B	158742950:A/C	TTCTATCTGATTTGCTTTA	TGAAACATTCCATACATCC	51	233	TaqI	233	68 + 165 + 233	68 + 165	[3]
rs1800925	IL13	131992809:C/T	GTCGCCTTTTCCTGCTCTTCCCGC	GGAATCCAGCATGCCTTGTGAGG	65	247	Bsh1236I	23 + 224	23 + 224 + 247	247	[4]
rs20541	IL13	131995964:C/T	TAGGCTGAAGACGGGCAGCA	AAGAAACTTTTTCGCGAGGG**C**C	63	199	MspI	22 + 177	22 + 177 + 199	199	[5]
rs56245420	IL15	142873720:A/T	TTTCTGTTATTAACAAACATCACTCTG	CAACACTTGTACATATTTTTATTCAA**T**AT	54	274	SspI	27 + 247	27 + 247 + 274	274	This study

^a^Mismatch is shown in bold and underlined font.

**Table 2:** Genotyping of seven studied SNPs in psoriasis patients (*n* = 200) and controls (*n* = 298).

SNP	Gene	Population	Genotype (%)			Minor allele (%)
rs2243250	IL4		T/T	T/C	C/C	C
		Controls	189 (63.4)	98 (32.9)	11 (3.7)	120 (20.1)
		Psoriasis	127 (63.5)	61 (30.5)	12 (6.0)	85 (21.3)

rs1800872	IL10		A/A	A/C	C/C	C
		Controls	138 (46.3)	123 (41.3)	37 (12.4)	197 (33.1)
		Psoriasis	93 (46.5)	86 (43.0)	21 (10.5)	128 (32.0)

rs3212227	IL12B		A/A	A/C	C/C	C
		Controls	119 (39.9)	128 (43.0)	51 (17.1)	230 (38.6)
		Psoriasis	77 (38.5)	104 (52.0)	19 (9.5)^∗a^	142 (35.5)

rs1800925	IL13		C/C	C/T	T/T	T
		Controls	222 (74.5)	72 (24.2)	4 (1.3)	80 (13.4)
		Psoriasis	140 (70.0)	52 (26.0)	8 (4.0)	68 (17.0)

rs20541	IL13		C/C	C/T	T/T	T
		Controls	146 (49.0)	126 (42.3)	26 (8.7)	178 (29.9)
		Psoriasis	100 (50.0)	80 (40.0)	20 (10.0)	120 (30.0)

rs56245420	IL15		A/A	A/T	T/T	T
		Controls	139 (46.6)	135 (45.3)	24 (8.1)	183 (30.7)
		Psoriasis	78 (39.0)	97 (48.5)	25 (12.5)	147 (36.8)

rs11209026	IL23R		G/G	G/A	A/A	A
		Controls	298 (100)	0	0	0
		Psoriasis	200 (100)	0	0	0

^∗a^ *P* = 0.0218.

**Table 3:** Correlation of psoriasis symptoms with SNPs in the IL genes.

SNP	Genotype	Psoriasis	Controls	OR (95% CI)	Allele	Psoriasis	Controls	OR (95% CI)
rs2243250	TT	127	189		T	315	476	
	TC	61	98	0.93 (0.63–1.37)	C	85	120	1.07 (0.78–1.46)
	CC	12	11	1.62 (0.69–3.78)				
	TC-CC	73	109	1.00 (0.69–1.45)				

rs1800872	AA	93	138		A	272	399	
	AC	86	123	1.04 (0.71–1.52)	C	128	197	0.95 (0.72–1.25)
	CC	21	37	0.84 (0.46–1.53)				
	AC-CC	107	160	0.99 (0.69–1.42)				

rs3212227	CC	19	51		C	142	230	
	AC	104	128	2.18 (1.21–3.92)	A	258	366	1.14 (0.88–1.48)
	AA	77	119	1.71 (0.96–3.17)				
	AC-AA	181	247	1.97 (1.12–3.45)				

rs1800925	CC	140	222		C	332	516	
	CT	52	72	1.15 (0.76–1.74)	T	68	80	1.32 (0.93–1.86)
	TT	8	4	3.17 (0.94–10.72)				
	CT-CC	60	76	1.25 (0.84–1.86)				

rs20541	CC	100	146		C	280	418	
	CT	80	126	0.93 (0.64–1.36)	T	120	178	1.01 (0.77–1.33)
	TT	20	26	1.12 (0.59–2.12)				
	CT-TT	100	152	0.96 (0.67–1.37)				

rs56245420	AA	78	139		A	253	413	
	AT	97	135	1.28 (0.87–1.87)	T	147	183	1.28 (1.00–1.71)
	TT	25	24	1.85 (0.99–3.46)				
	AT-TT	122	159	1.36 (0.94–1.96)				
